# A pilot study of micro-CT-based whole tissue imaging (WTI) on endoscopic submucosal dissection (ESD) specimens

**DOI:** 10.1038/s41598-022-13907-6

**Published:** 2022-06-14

**Authors:** Hirotsugu Sakamoto, Makoto Nishimura, Alexei Teplov, Galen Leung, Peter Ntiamoah, Emine Cesmecioglu, Noboru Kawata, Takashi Ohnishi, Ibrahim Kareem, Jinru Shia, Yukako Yagi

**Affiliations:** 1 Department of Pathology, Memorial Sloan Kettering Cancer Center, MSKCC Josie Robertson Surgery Center, 1133 York Ave. Suite 1020, New York, NY 10065 USA; 2 Department of Medicine, Division of Gastroenterology, Jichi Medical University, Shimotsuke, Japan; 3 Gastroenterology, Hepatology and Nutrition Service, Memorial Sloan Kettering Cancer Center, New York, NY USA; 4 Division of Endoscopy, Shizuoka Cancer Center, Shizuoka, Japan

**Keywords:** Colorectal cancer, Gastric cancer, Colorectal cancer, Gastric cancer

## Abstract

Endoscopic submucosal dissection can remove large superficial gastrointestinal lesions in en bloc. A detailed pathological evaluation of the resected specimen is required to assess the risk of recurrence after treatment. However, the current method of sectioning specimens to a thickness of a few millimeters does not provide information between the sections that are lost during the preparation. In this study, we have produced three-dimensional images of the entire dissected lesion for nine samples by using micro-CT imaging system. Although it was difficult to diagnose histological type on micro-CT images, it successfully evaluates the extent of the lesion and its surgical margins. Micro-CT images can depict sites that cannot be observed by the conventional pathological diagnostic process, suggesting that it may be useful to use in a complementary manner.

## Introduction

Endoscopic submucosal dissection (ESD) is an endoscopic treatment for large superficial gastrointestinal (GI) lesions that are difficult to remove en bloc by conventional endoscopic mucosal resection^1^. In recent years, it has rapidly spread throughout the world including the United States^2^. Lesions resected en bloc by ESD are subject to a detailed pathological evaluation. After they are sectioned a few millimeters thick then approximately 10 or more fixed paraffin embedded blocks are made, Hematoxylin and Eosin (H&E) stained slides are created and the risk of subsequent recurrence is assessed by findings such as tumor type, margin of the lesion, depth of invasion, lymphovascular invasion, and tumor budding^3,4^. However, the slides produced represent only a small cross-section of the lesion, and there is always the possibility of missing significant findings in other areas. In fact, a multicenter study in North America reported that residual recurrence after R0 resection (no lesion extended to resection margins on pathological evaluation) was observed in 5.8% of patients^2^.

Whole tissue imaging (WTI) and whole block imaging (WBI) by micro-CT is a novel in vitro tomographic method allowing examination of fresh or fixed tissues and formalin-fixed paraffin-embedded (FFPE) blocks in a non-destructive manner with a spatial resolution up to the level of 1 µm^5–8^. Previous studies for breast cancer suggested that micro-CT images of fresh specimens showed structures identified vessel network, metastatic tumor, and lymph node tissue^5,6^. Moreover, in our previous studies, we have reported that micro-CT images of FFPE blocks were able to detect capsular invasion, vascular invasion, and assess the volume of nodal metastasis in thyroid carcinoma without tissue sectioning^8^. To avoid the elaborate process of making FFPE blocks as well as the long scanning times of these, we have developed a method to evaluate fresh specimens by Micro-CT scanning in a short time after immersion in Lugol's iodine solution as a method that can be applied to practical clinical practice and have reported a case of micro-CT scanning of an ESD specimen of early-stage rectal cancer^9^. However, there are no reports comparing micro-CT images of superficial GI lesions with hematoxylin and eosin-stained slides. The aim of this study is to evaluate the utility of micro-CT for pathological evaluation of superficial GI lesions resected by ESD without any delay in the routine pathology diagnosis process.

## Results

**Figure 1:**
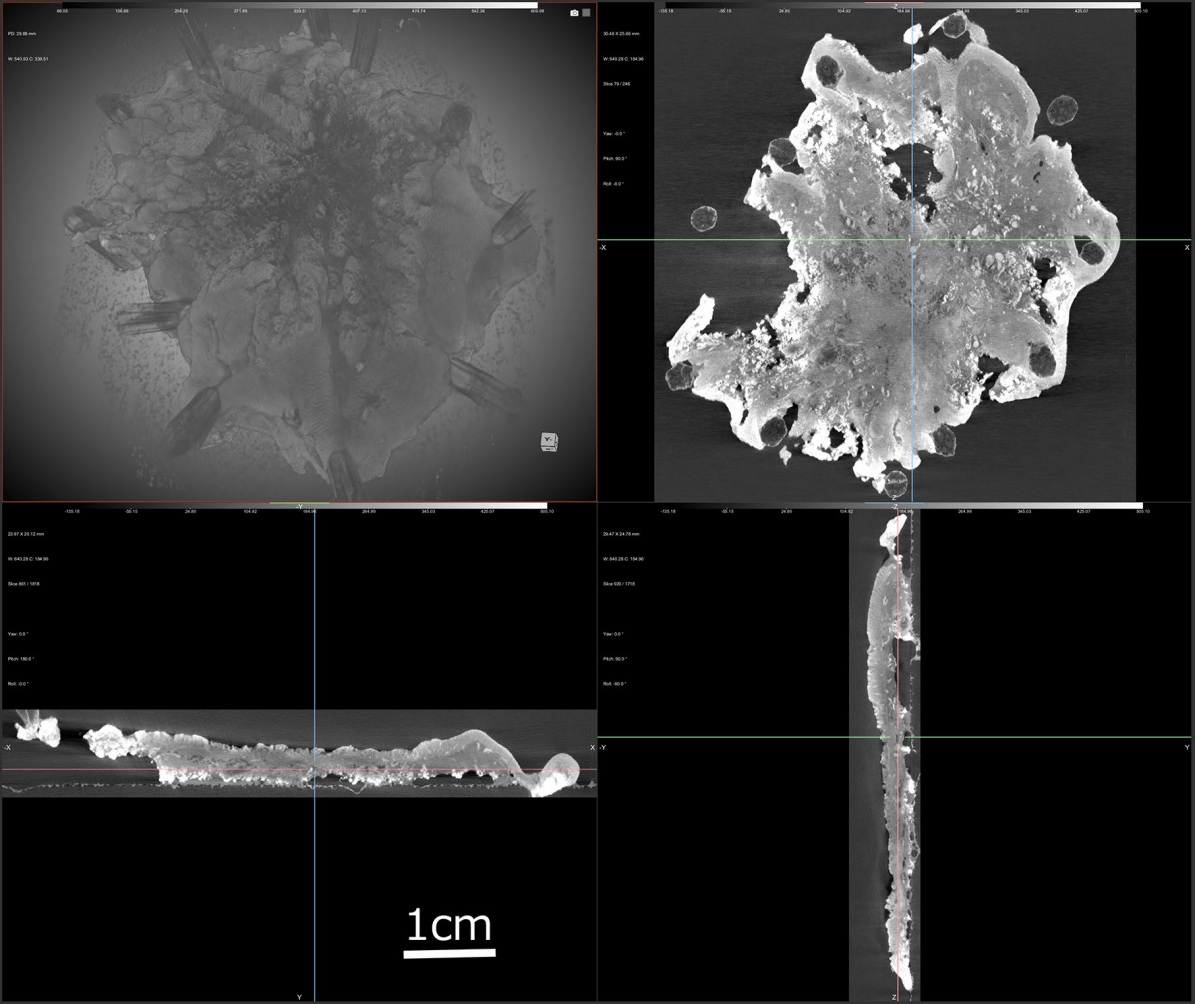
Whole tissue imaging (WTI) of case 5 using micro-CT. Top left; 3D image, bottom left, top right, bottom right; 2D images of different cross sections. WTIs obtained from Micro-CT scans can produce images of arbitrary cross sections.

**Figure 2:**
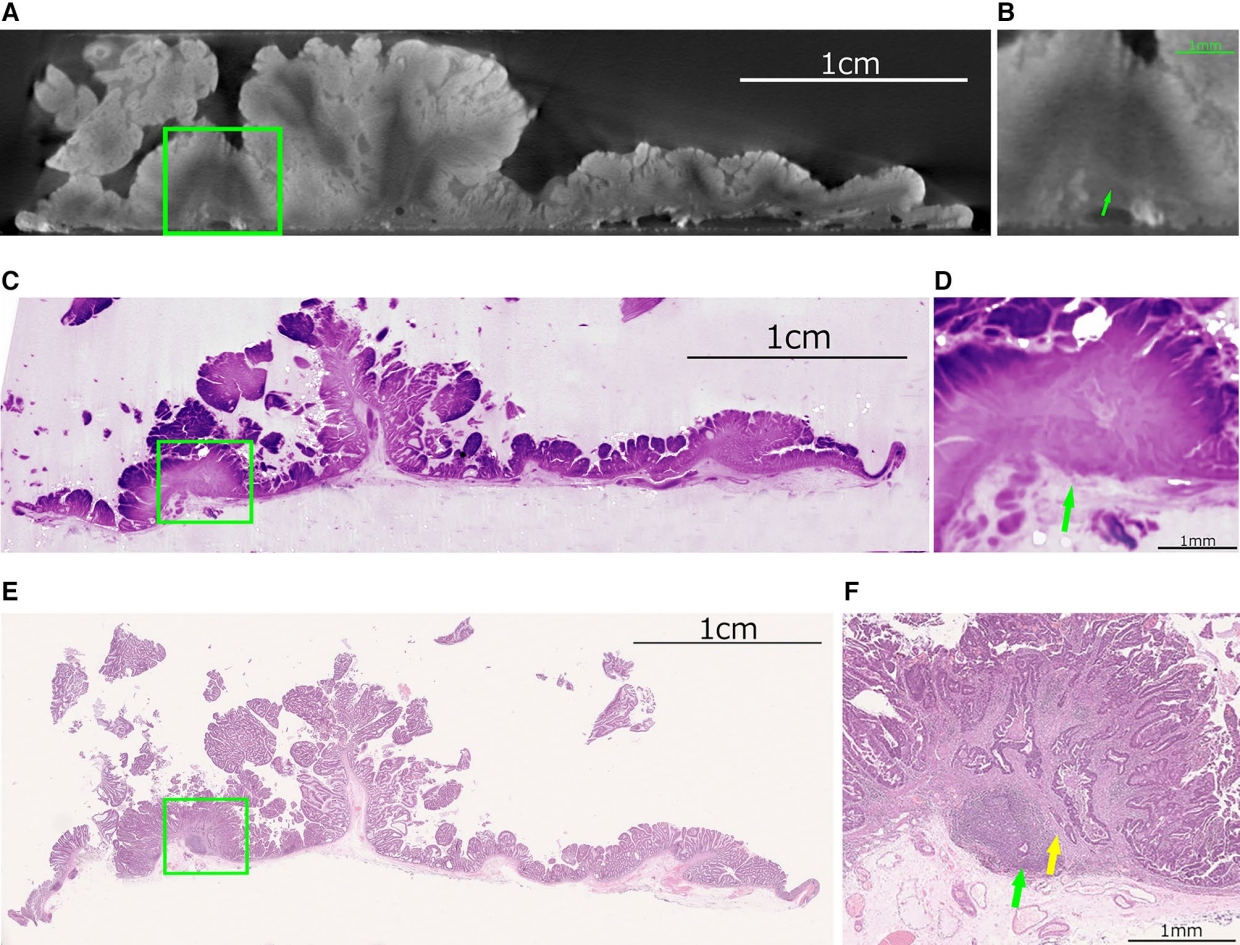
Images of the submucosal invasion site (green arrow) of case 6. (**a**) (Low-power field) (**b**) (High-power field) Whole tissue image (WTI). (**c**) (Low-power field) d. (high-power field) Whole block image (WBI). (**e**) (Low-power field) (**f**) (High-power field) Whole slide image (WSI). WTI and WBI were able to confirm the submucosal invasion diagnosed by WSI, but WTI and WBI were unable to recognize the lymphatic invasion site diagnosed by WSI (yellow arrow).

**Figure 3:**
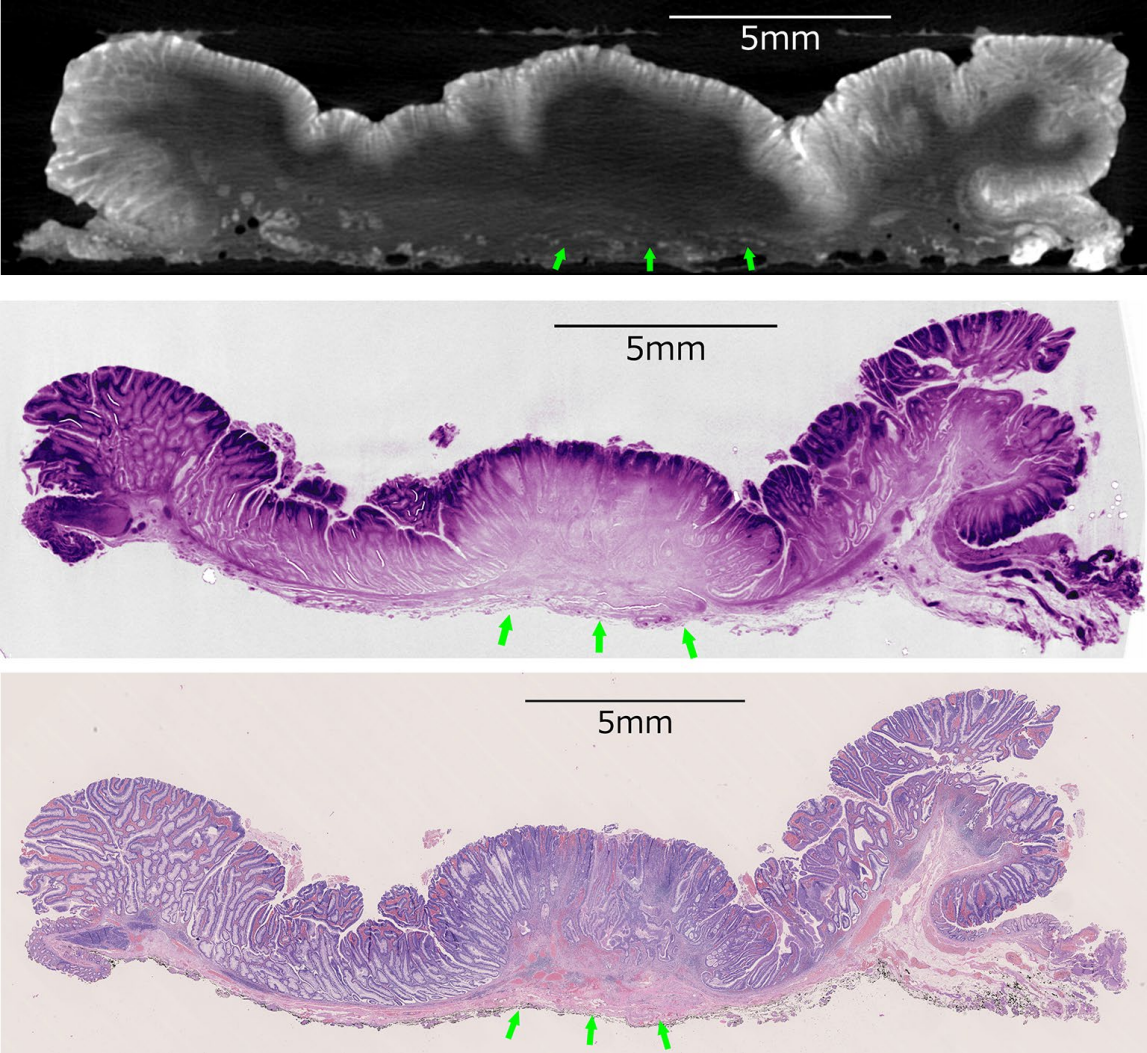
Images of the submucosal invasion site (arrow) of case 9. (**a**) Whole tissue image (WTI). (**b**) Whole block image (WBI). (**c**) Whole slide image (WSI). WBI was able to confirm the submucosal invasion diagnosed by WSI, but WTI was unable to recognize the submucosal invasion site due to partial staining failure.

One gastric intramucosal cancer, 3 colorectal (1 intramucosal, 2 submucosal) cancer, and 5 colorectal adenomas were scanned by micro-CT (Table 1). There was no effect of immersion in 10% Lugol's solution on the quality of the H&E stained slides. We were able to obtain evaluable WTIs for all nine specimens, and the WTIs allowed us to create 2-Dimensional images of any cross-section along with 3-Dimensional images (Fig. 1, Supplementary Fig. S1, with annotations, Supplementary Video 1, which demonstrates the 3-Dimensional video). In the images of the specimens obtained by WTI, all of the lesions were clearly different from the surrounding normal mucosa and showed dense glandular ducts or tall glandular structures similar to those observed in WSIs. We were able to point out the extent of the lesion using WTIs, and we were also able to evaluate the deep margins (whether the lesion is present on the deep edge of the resection specimen) and lateral margins (whether the lesion is present on the lateral edge of the resection specimen) of each specimen. However, it was not possible to make a qualitative diagnosis such as adenoma, high-grade dysplasia, or adenocarcinoma for each lesion because it was difficult to confirm the structure of individual cells on WTI. In fact, four of the six (67%) lesions with high-grade dysplasia or adenocarcinoma showed irregular ductal structures of unequal size on WTI, whereas a qualitative diagnosis could not be made because of the difficulty in assessing nuclear atypia. Additionally, the contrast effect was relatively good in the shallow mucosal layer and submucosa, but 78% (7/9) of lesions had poor contrast effects in the deep mucosal layer. In two lesions with submucosal invasion, the contrast effects in the deep mucosal layer were also poor. In one lesion, the submucosal invasion was confirmed by WTI (Case 6, Fig. 2, Supplementary Fig. S2, with annotations), but in another lesion, the submucosal invasion was difficult to detect by WTI (Case 9, Fig. 3, Supplementary Fig. S3, with annotations, Supplementary Video 2, which demonstrates the merged video of WTI, WBI, and whole slide image (WSI)).

**Table 1:** Characteristics of the lesions.

	Age (years)	Gender	Location	Morphology	Maximum diameter (mm)	Diagnosis	Lateral margin (WSI/WTI/WBI)	Deep margin (WSI/WTI/WBI)	Areas where it is difficult to confirm the internal structure due to poor staining in WTI
1	54	F	Cecum	0-IIa	18	Intramucosal AC arising in TA with HGD	−/−/−	−/−/−	+
2	82	M	Hepatic flexure	0-IIa + Is	22	TA	−/−/−	−/−/−	+
3	61	F	Rectum	0-Is	35	TVA with HGD	−/−/−	−/−/−	+
4	54	F	Rectum	0-IIa	41	TVA	−/−/−	−/−/−	+
5	63	F	Transverse colon	0-IIa + IIc	25	TA	−/−/−	−/−/−	−
6	51^a^	F	Rectum	0-IIa	45	submucosal AC arising in TVA with HGD	+ (adenoma)/ + / +	−/−/−	+
7	63	M	Rectum	0-IIa + IIc	40	TSA with HGD	−/−/−	−/−/−	+
8	72	M	Stomach, antrum	0-IIc	31	Intramucosal AC	−/−/−	−/−/−	−
9	67	F	Ascending colon	0-IIa	45	Submucosal AC arising in TVA with HGD	+ (adenoma)/+/+	−/−/−	+

*WSI* whole slide imaging, *WTI* whole tissue imaging, *WBI* whole block imaging, *AC* adenocarcinoma, *TA* tubular adenoma, *HGD* high grade dysplasia, *TVA* tubilovillous adenoma, *TSA* traditional serrated adenoma.

^a^A summary of case 6 is reported^9^.

We were able to obtain more detail with clearer delineated images with WBI compare to WTI. As with WTI and WBI, we were able to assess tumor extent and margins but had difficulty qualitatively assessing the lesion. Pathological diagnosis revealed lymphatic invasion in Case 6, but it was difficult to identify this on WBI. However, since no findings suggestive of lymphatic vessel invasion could be confirmed in the H&E slide prepared after WBI acquisition, it is considered that the lymphatic invasion site was entirely resected when the first slides were made (Fig. 2). Even with WTI, it was difficult to point out lymphatic invasion at the same site. The sections made by WSI were otherwise all confirmed on WTI and WBI. In this study, we did not find additional pathologic features by WTI and WBI that were not identified on WSI.

## Discussion

To the best of our knowledge, this is the first case series in which ESD specimens were scanned and evaluated using micro-CT. The WTI and WBI obtained from the micro-CT scan, while incomplete, were able to confirm the detailed tissue structures and point out the extent of each lesion. Since the image resolution of micro-CT is defined by the distance between the source and the specimen^10^, ESD specimens, which are smaller than surgical specimens of GI cancers, were considered to be suitable for evaluation by micro-CT imaging.

Detailed pathological examination of superficial GI lesions resected en bloc by ESD is important for assessing the risk of subsequent recurrence, especially whether R0 resection has been achieved, which is essential for assessing the risk of residual recurrence^11–13^. However, since the pathological diagnosis is based on slides made every few millimeters of ESD resected specimen, it is difficult to completely exclude the possibility that a positive margin was present in a section where no slides were made. In this study, both WTI and WBI were able to assess margins in all specimens but did not find additional information that might change the diagnosis made with WSI.

The distance of submucosal invasion is also an important finding in assessing the risk of recurrence. When the submucosal invasion is more than 500 μm in gastric cancer, and more than 1000 μm in colorectal cancer, the risk of lymph node metastasis increases and additional surgery is recommended^11–13^. Diagnosis of submucosal invasion based on surface structure is mainly done by magnified observation with an endoscope^14,15^, but it is not easy to accurately identify the deepest invasion site in a specimen and to make an H&E slide of its cross-section. In fact, cases of lymph node metastasis recurrence despite the diagnosis of R0 resection of intramucosal carcinoma after ESD has been reported. This suggests that submucosal invasion existed in the section where H&E slides were not made^16,17^. WTI and WBI with micro-CT scans can observe findings that cannot be confirmed by conventional pathology slides and may lead to a more accurate diagnosis. However, in one of the two lesions in which submucosal invasion was observed by WSI, the invasion site was pointed out on WBI but could not be clearly identified on WTI. The major reason for this was that the deep part of the mucosal layer was poorly stained.

Evaluation of the presence of lymphatic and vascular invasion is also essential for pathological diagnosis of ESD specimens^11–13^. In the specimen examined in this study, the lymphatic invasion was observed in one lesion at the time of pathological diagnosis, but the area where the lymphatic invasion was observed did not remain in the WBI performed afterward, and the lymphatic invasion could not be confirmed in the WSI prepared after WBI imaging. In addition, in submucosal invasive colorectal cancer, the risk of lymph node metastasis recurrence is also evaluated by the presence or absence of budding^11–13^, but there was no specimen with budding on WSI in this study. Therefore, it is necessary to collect specimens with lymphatic invasion, vascular invasion, and budding for further analysis. Moreover, in order to objectively evaluate the usefulness of micro-CT imaging, a comparative study in which H&E and Micro-CT images are evaluated blindly is necessary.

Next to Lugol's solution, several other contrast agents have been reported to be candidates for micro-CT^18^. Müller et al. reported that a hematein-based X-ray staining method enables micro-CT imaging of cell nuclei in mouse liver^19^. However, this method is difficult to use for clinical specimens at this stage because it requires about 10 days for staining and cannot depict structures other than the nucleus. Therefore, Micro-CT cannot completely replace the conventional pathological diagnosis by H&E slides. However, since it is thought to be possible to accurately grasp the extent of the tumor, it is expected that the deepest part of the tumor can be evaluated by WTI before sectioning the specimen and that a higher quality pathological diagnosis can be made by sectioning that specific area for H&E slide. To achieve this, it is necessary to produce a clear WTI with no staining defects. Xia et al. reported that favorable Micro-CT images were obtained by staining tongue squamous cell carcinoma specimens with 3% Lugol's iodine solution for 12 h^20^. The ESD specimens used in this study were collected in routine clinical practice, and we cannot perform any protocol that would affect the conventional pathological diagnosis. In a study using test samples before the start of this study, 10% Lugol's iodine solution caused unacceptable damage to the specimens when staining was performed for more than 180 s, so it was difficult to extend the time further. Prolonged staining with diluted Lugol’s iodine solution was also difficult because it would have prolonged the time to pathological diagnosis. However, some specimens could not be evaluated sufficiently with the staining method used in this study, and the staining method needs to be improved. It is also necessary to evaluate the effect of staining with Lugol’s iodine solution on immunostaining. Although it has been reported that staining with Lugol’s iodine solution does not affect immunostaining for neuronal-related proteins^21^, we have experienced poor immunostaining for mismatch repair proteins due to staining with Lugol’s iodine solution.

If the method of WTI by micro-CT scan in ESD specimen is established, it can be applied to other specimens. It may be possible to derive a more accurate pathological diagnosis by clarifying the most important cross-section by WTI before sectioning. Furthermore, since a much larger number of micro-CT images can be produced per specimen compared to conventional pathology images, it can save labor in the evaluation of obtaining images for clinical application. Ongoing development of artificial intelligence technologies has the potential to develop micro-CT image interpretation support systems that directly lead to pathological diagnosis by combining deep machine learning with biomedical image processing algorithms such as texture matching and non-rigid registration algorithms^22–24^.

In conclusion, WTI and WBI using micro-CT could delineate the extent of the lesion in ESD specimens. Thus, WTI and WBI have the potential to compensate for the limitations of conventional pathological diagnosis and help reduce the labor required for pathological diagnosis. However, improvement of image quality is essential for the diagnosis of ESD specimens with micro-CT in clinical practice.

## Methods

We selected nine GI lesions that were resected en bloc by ESD at Memorial Sloan Kettering Cancer Center between October 2020 and December 2020 for inclusion. Written informed consent for ESD was obtained from all patients. This study was approved by the Institutional Review Board of Memorial Sloan Kettering Cancer Center (20-076). All patients provided written informed consent for the use of pathology specimens for research purposes. The authors confirm that all research was performed in accordance with relevant guidelines/regulations. The research was performed in accordance with the Declaration of Helsinki.

### Staining optimization

Soft biological tissue such as the gastrointestinal tract lack the high X-ray contrast properties which are inherent to hard tissue such as bone or teeth. In order to enhance contrast in the tissues, we have stained them with Lugol's iodine solution. Iodine's semimetallic properties have been shown to enhance nonhuman soft-tissue X-ray contrast and enable the high-resolution visualization of minute morphological details^25^.

Prior to starting the study, we have conducted a series of staining optimization experiments using 10 test tissues. In our research, our entire processing time of staining and scanning was up to 30 min because not delay the rest of the pathology diagnosis process. The estimated scanning duration for most tissues is 15 min.

We have modified concentration (1–10%) and duration (1–15 min) to optimize the staining protocol (see Supplementary Fig. S4 online). We have decided to use a 10% Lugol's iodine solution for 60 s at the start of this study. However, we expected that we have to modify the parameters slightly during the study because enhanced results of the same organ are not always the same by the tissue size, tissue density and so on.

### Whole tissue imaging

The specimens resected by ESD were immediately immersed in 10% Lugol's iodine solution as a contrast medium for 60–180 s, except for one specimen that was fixed in 10% formalin because it could not be scanned immediately. They were then stretched, resection side down, on extruded polystyrene foam boards with ultrathin bamboo picks, and the sides and front were also covered with extruded polystyrene foam boards to prevent drying. They were immobilized with a carbon fiber paddle and immediately scanned for 10–15 min with a custom-made micro-CT scanner (Nikon XT H 160 MedX Alpha, Nikon Metrology NV, Leuven, Belgium). The scan parameters were shown in Table 2. Scanned image slices reconstructed using modified Feldkamp filtered back projection algorithms with CTPro3D XT 5.1.4.2 MedX 1 (Nikon Metrology NV). The micro-CT images of the fresh and fixed specimens were defined WTIs in this study. Supplementary Fig. S5 shows an example of visualized resolution comparison between WTI and WSI. Thereafter, the specimens were directly infiltrated in 10% formalin for fixation. After the fixed specimens were sectioned, photographs were taken to show the incision sites. The sectioned specimens were embedded in paraffin blocks. Each block was thin-sliced and glass slides were prepared for pathological diagnosis. Pathological diagnoses, such as qualitative diagnosis of adenoma, high-grade dysplasia, and adenocarcinoma, as well as diagnosis of extent and depth of the lesions, were made based on the 8th edition of the AJCC Cancer Staging Manual^26^.

**Table 2:** Acquisition parameters of micro-CT.

Object	Beam energy (kV)	Beam current (µA)	Exposure time (ms)	Projections, n	Frames per projection	Source-to-object distance (mm)	Resolution (µm)
Fresh and fixed tissue	90	133	250	1201–2401	1–2	60.8–149.5	14.3–31.5 Ave. 27.4
FFPE block	70	100	3250	4821	4	16.8–109.4	9.4–19.7 Ave. 12.88

FFPE, formalin fixed, paraffin embedded; Micro-CT, micro-computed tomography; msec, milliseconds.

### Whole block imaging and whole slide imaging

In the course of the pathological diagnosis, a total of 77 FFPE blocks were obtained for these nine specimens. After the pathology report was signed out, each FFPE block was immobilized with a carbon fiber paddle and scanned with micro-CT in the same way as the fresh specimens, with different scanning time (21 h and 50 min) and parameters as shown in Table 2. The micro-CT images of the FFPE blocks were defined WBIs in this study.

After obtaining the WBI, each FFPE block was thinly sliced again to create H&E stained slides in order to obtain the same histological images as the cross-section images of the WBIs. They were scanned by a whole-slide scanner (NanoZoomer 2.0-HT or 60S, Hamamatsu Photonics K.K., Shizuoka, Japan) at 20× magnification (0.46 micron/pixel). The resulting images were termed WSIs.

### Evaluation of images

The reconstructed imaging data of WTIs and WBIs were visualized and analyzed by using 3D imaging software such as VG Studio MAX 2.2.6 (Volume Graphics GmbH, Heidelberg, Germany), myVGL 3.4.2 (Volume Graphics GmbH) and Dragonfly 2020 (Object Research Systems, Montreal, Quebec, Canada). The WBI images were adjusted to be similar to the H&E stain by changing the color manually according to the density of each region obtained from the Micro-CT scan using VG Studio MAX 2.2.6. NDP.view 2.8.24 (Hamamatsu Photonics K.K.) was used to reference the WSIs. After reading the extent of lesions for each image of WTIs, WBIs, and WSIs, we compared images of the same section whenever possible to see if WTIs and WBIs produced findings comparable to those seen in WSIs. The positions of WTI, WBI, and WSI were compared based on the images taken when the specimens were sectioned. If the lesion was carcinoma, the presence and degree of submucosal invasion and vascular invasion were confirmed by WSI, and the same findings were examined by WTI and WBI. In all specimens, we also evaluated whether 3D images by WTI and WBI can identify pathologic features other than what was found in sections created for WSI.

## Supplementary Information

Supplementary Legends.

Supplementary Figure S1.

Supplementary Figure S2a.

Supplementary Figure S2b.

Supplementary Figure S2c.

Supplementary Figure S2d.

Supplementary Figure S2e.

Supplementary Figure S2f.

Supplementary Figure S3a.

Supplementary Figure S3b.

Supplementary Figure S3c.

Supplementary Figure S4a.

Supplementary Figure S4b.

Supplementary Figure S4c.

Supplementary Figure S4d.

Supplementary Figure S4e.

Supplementary Figure S5a.

Supplementary Figure S5b.

Supplementary Video 1.

Supplementary Video 2.

## Data Availability

The datasets generated during and/or analyzed during the current study are not publicly available due to clinical study but are available from the corresponding author on reasonable request.
