# Supplementary material for: A pilot study of micro-CT-based whole tissue imaging (WTI) on endoscopic submucosal dissection (ESD) specimens
Source: Sci Rep. 2022 Jun 14;12:9889. doi: 10.1038/s41598-022-13907-6 (PMC9198046; doi:10.1038/s41598-022-13907-6)
Supplement: Supplementary file 1 — Supplementary Legends. [file 41598_2022_13907_MOESM1_ESM.docx]

**Supplemantary figure and video legends**

**Supplementary Fig. S1** Whole tissue imaging (WTI) of case 5 using micro-CT with annotations. Top left; 3D image, bottom left, top right, bottom right; 2D images of different cross sections.

WTIs obtained from Micro-CT scans can produce images of arbitrary cross sections. The location of the mucosal layer was indicated by light blue brackets and the location of the submucosal layer by yellow brackets. The extent of the lesion was surrounded by a green line.

**Supplementary Fig. S2** Images of the submucosal invasion site (green arrow) of case 6 with annotations. **a.** (low-power field) **b.** (high-power field) Whole tissue image (WTI). **c.** (low-power field) **d.** (high-power field) Whole block image (WBI). **e.** (low-power field) **f.** (high-power field) Whole slide image (WSI).

The location of the mucosal layer was indicated by light blue brackets and the location of the submucosal layer by green brackets. The extent of the lesion was surrounded by a yellow line. WTI and WBI were able to confirm the submucosal invasion diagnosed by WSI, but WTI and WBI were unable to recognize the lymphatic invasion site diagnosed by WSI (yellow arrow). Light blue arrows indicated small arteries.

**Supplementary Fig. S3** Images of the submucosal invasion site (green arrow) of case 9 with annotations. **a.** Whole tissue image (WTI). **b.** Whole block image (WBI). **c.** Whole slide image (WSI).

The location of the mucosal layer was indicated by light blue brackets and the location of the submucosal layer by green brackets. The extent of the lesion was surrounded by a yellow line. WBI was able to confirm the submucosal invasion diagnosed by WSI, but WTI was unable to recognize the submucosal invasion site due to partial staining failure.

**Supplementary Fig. S4** Images obtained by different staining protocols with Lugol's iodine solution for normal human colon samples taken in part from colon cancer surgical specimens. **a.** No staining: The internal structure could not be discerned at all.. **b.** Staining with 1% Lugol's iodine solution for 30 seconds: The mucosal layer (light blue arrow) was slightly visible but indistinct. **c.** Staining with 10% Lugol's iodine solution for 30 seconds: The mucosal layer (light blue arrow) was slightly visible but indistinct. The muscular layer (yellow arrow) was also slightly visible. **d.** Staining with 10% Lugol's iodine solution for 1 minute: The mucosal (light blue arrow), submucosal (yellow arrow), and muscular layers (green arrow) could each be clearly identified. **e.** Staining with 10% Lugol's iodine solution for 3 minutes: The mucosal (light blue arrow), submucosal (yellow arrow), and muscular layers (green arrow) could each be clearly identified. However, some staining defects were observed in the center of the muscle layer (pink arrow).

All normal human colon tissues were scanned with micro-CT for 15 minutes. The resolutions were 15-18 um.

**Supplementary Fig. S5** Example images of visualized resolution comparison between Whole Tissue Image (WTI) and Whole Slide Image (WSI) of a test sample of advanced rectal cancer. **a.** 7um/voxel of WTI. **b.** 0.29x (17um/pixel) of WSI. Cancer areas (yellow arrows) were observed in both images. The cancer had invaded the muscularis propria (green arrows).

**Supplementary Video 1** Whole tissue imaging of case 5 using micro-CT. (One tick on the grid in the video represents 5 mm.)

WTIs obtained from Micro-CT scans can produce images of arbitrary cross sections.

**Supplementary Video 2** Comparison of whole tissue image (WTI), whole block image (WBI) and whole slide image (WSI) of the same section in case 9.
