# Supplementary figures and images for: A pilot study of micro-CT-based whole tissue imaging (WTI) on endoscopic submucosal dissection (ESD) specimens
Source: Sci Rep. 2022 Jun 14;12:9889. doi: 10.1038/s41598-022-13907-6 (PMC9198046; doi:10.1038/s41598-022-13907-6)

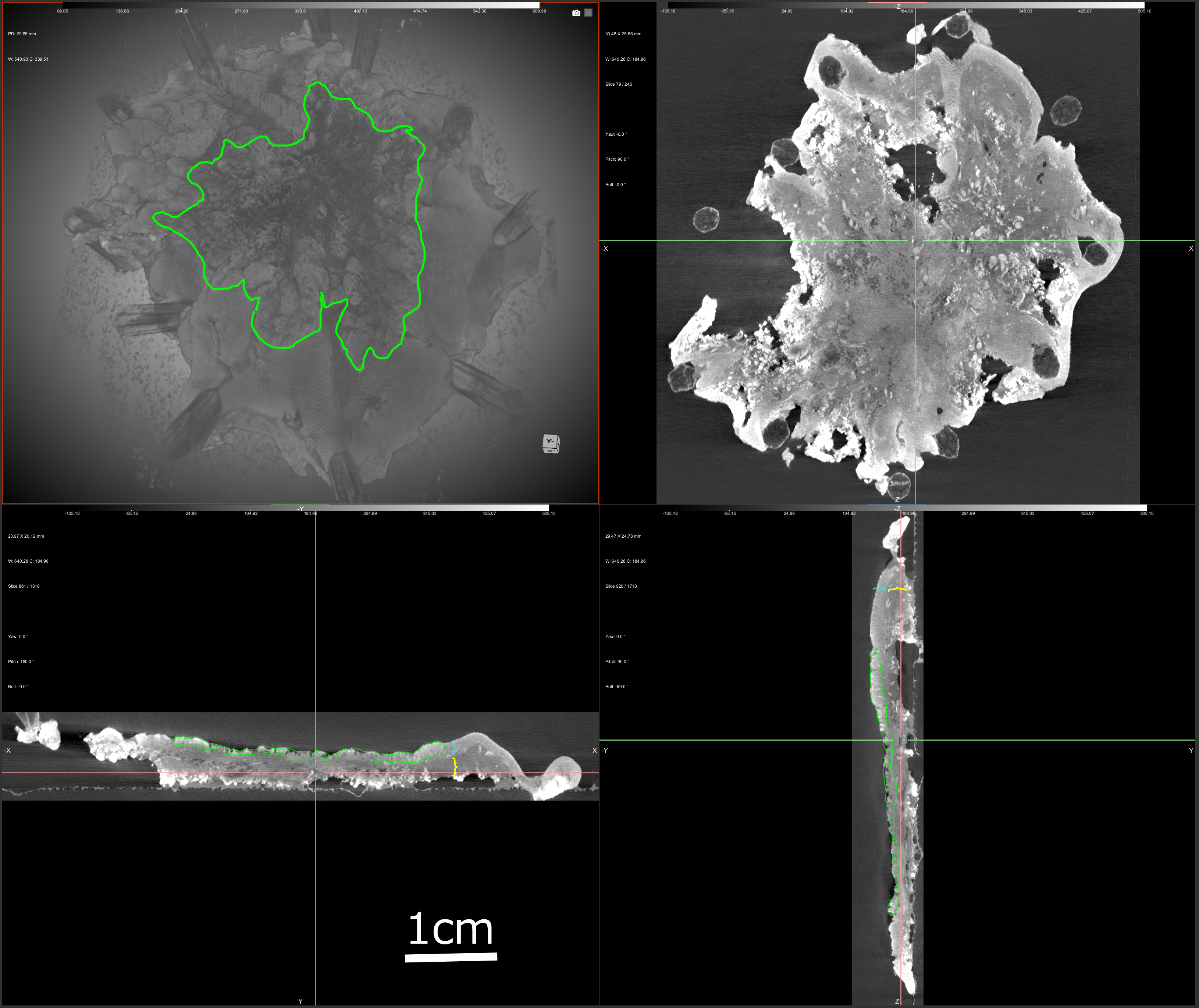

Supplement: Supplementary file 2 — Supplementary Figure S1. [file 41598_2022_13907_MOESM2_ESM.jpg]

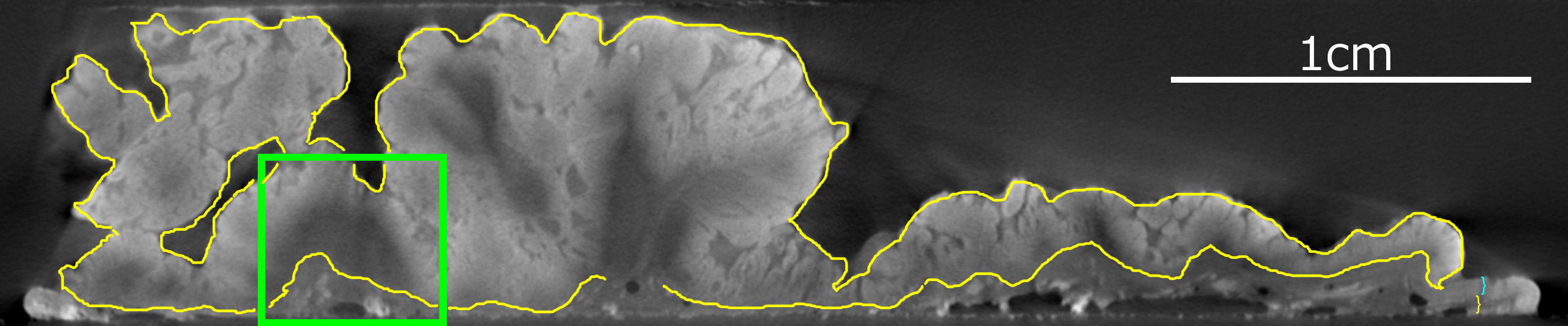

Supplement: Supplementary file 3 — Supplementary Figure S2a. [file 41598_2022_13907_MOESM3_ESM.jpg]

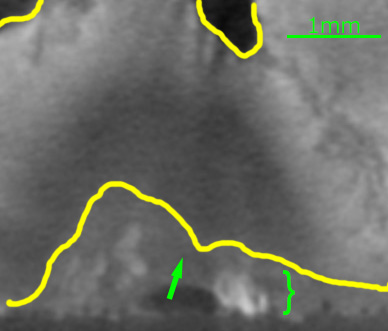

Supplement: Supplementary file 4 — Supplementary Figure S2b. [file 41598_2022_13907_MOESM4_ESM.jpg]

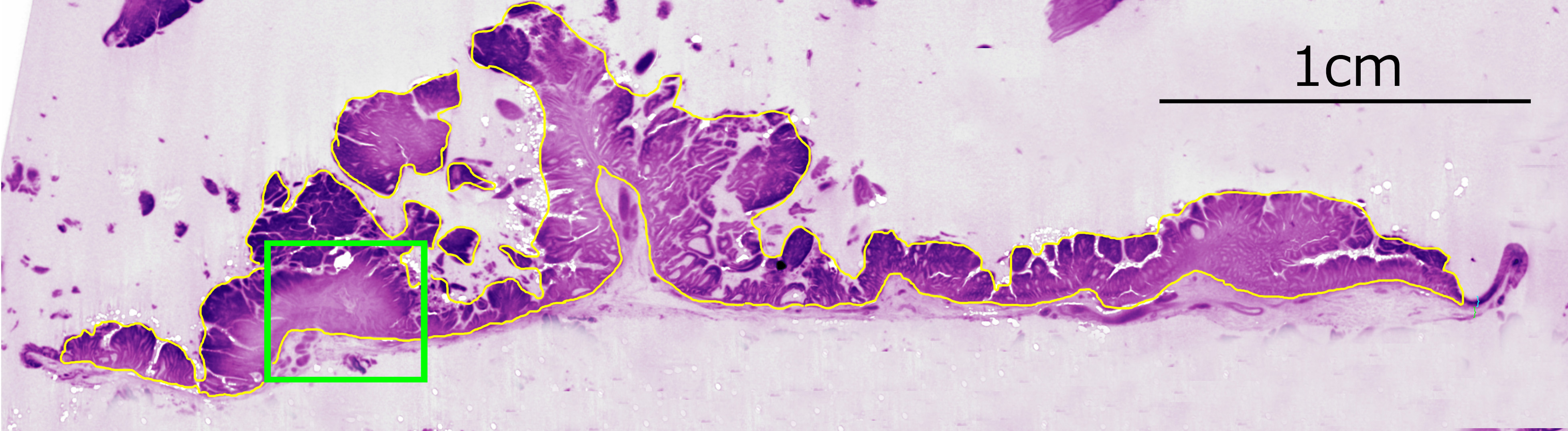

Supplement: Supplementary file 5 — Supplementary Figure S2c. [file 41598_2022_13907_MOESM5_ESM.jpg]

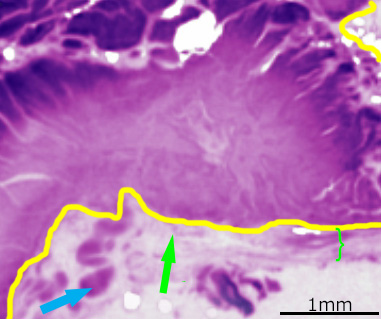

Supplement: Supplementary file 6 — Supplementary Figure S2d. [file 41598_2022_13907_MOESM6_ESM.jpg]

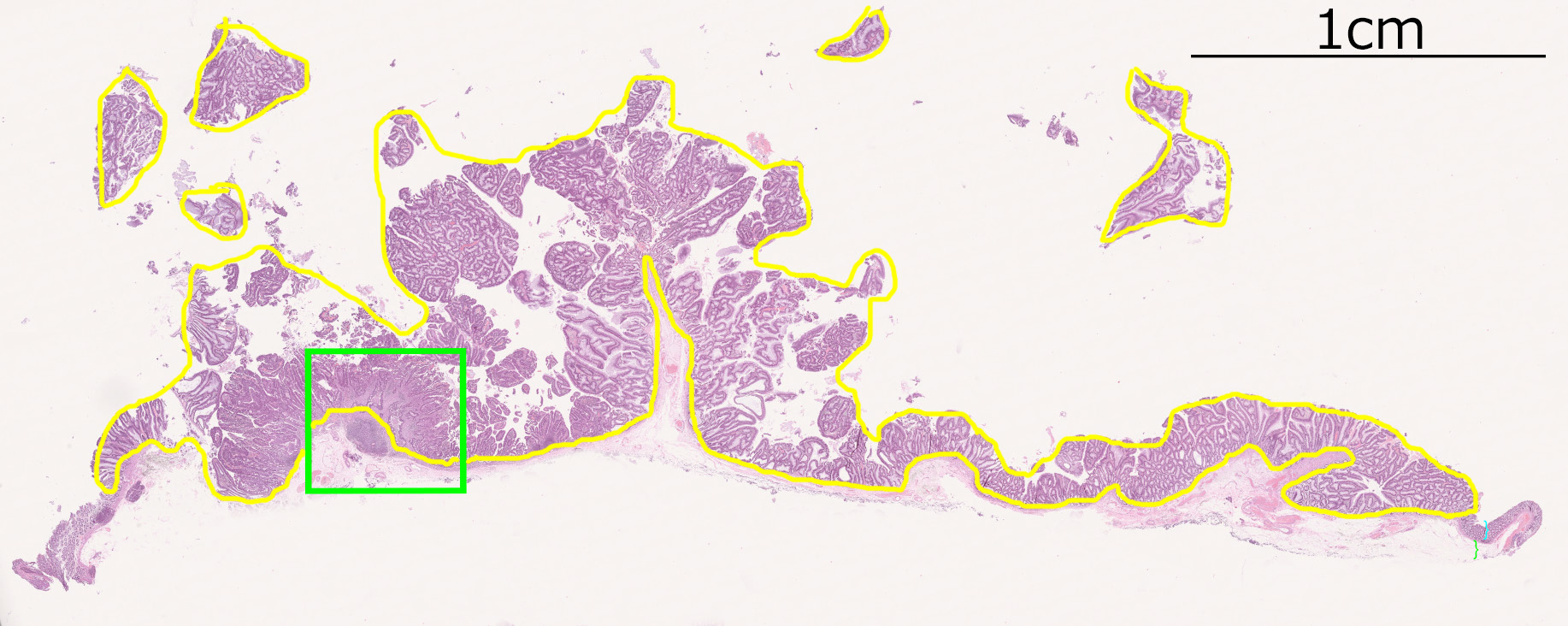

Supplement: Supplementary file 7 — Supplementary Figure S2e. [file 41598_2022_13907_MOESM7_ESM.jpg]

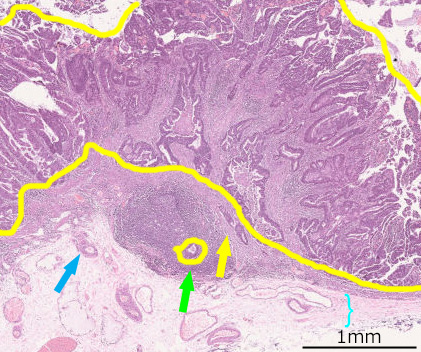

Supplement: Supplementary file 8 — Supplementary Figure S2f. [file 41598_2022_13907_MOESM8_ESM.jpg]

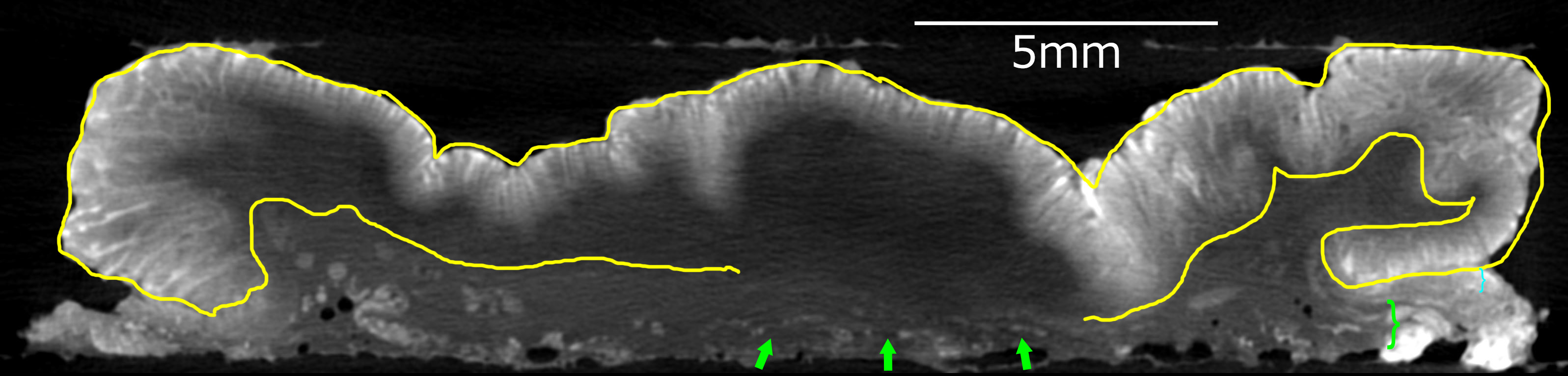

Supplement: Supplementary file 9 — Supplementary Figure S3a. [file 41598_2022_13907_MOESM9_ESM.jpg]

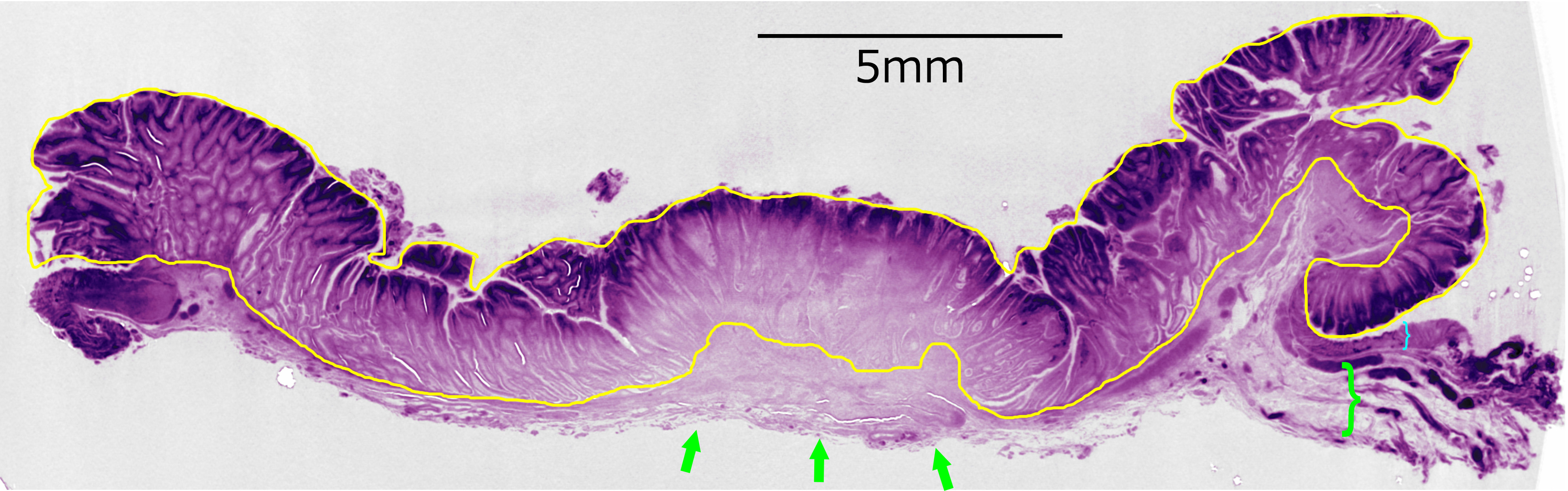

Supplement: Supplementary file 10 — Supplementary Figure S3b. [file 41598_2022_13907_MOESM10_ESM.jpg]

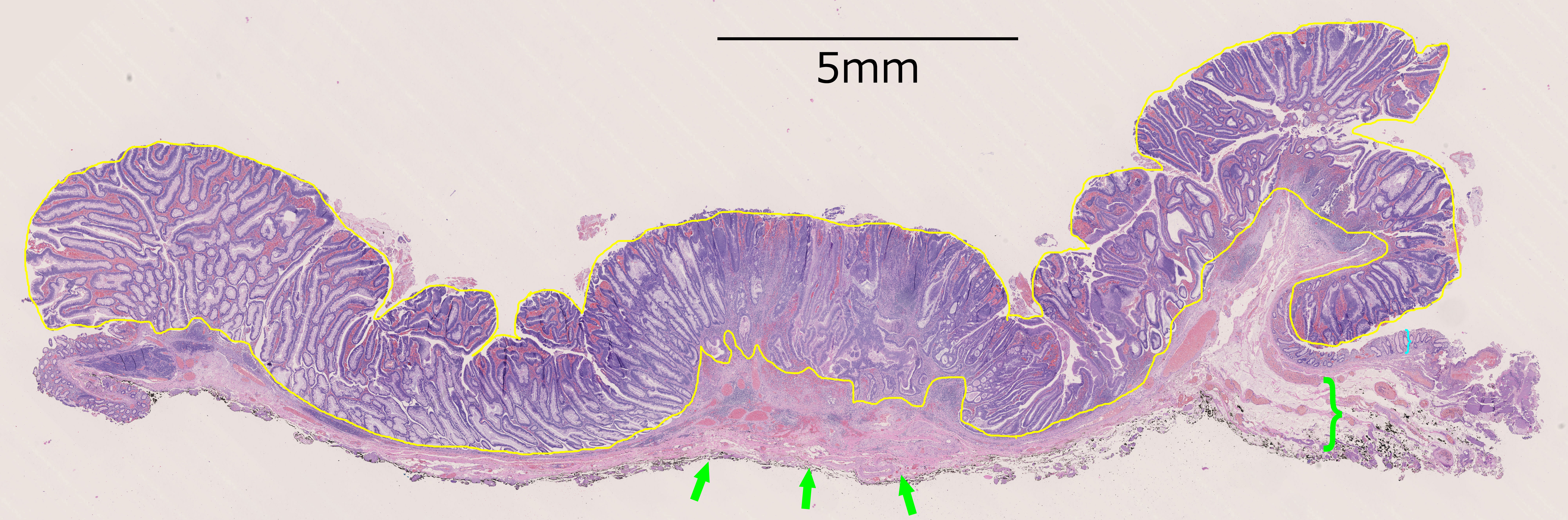

Supplement: Supplementary file 11 — Supplementary Figure S3c. [file 41598_2022_13907_MOESM11_ESM.jpg]

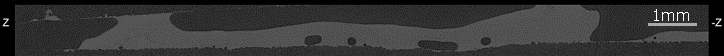

Supplement: Supplementary file 12 — Supplementary Figure S4a. [file 41598_2022_13907_MOESM12_ESM.jpg]

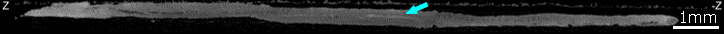

Supplement: Supplementary file 13 — Supplementary Figure S4b. [file 41598_2022_13907_MOESM13_ESM.jpg]

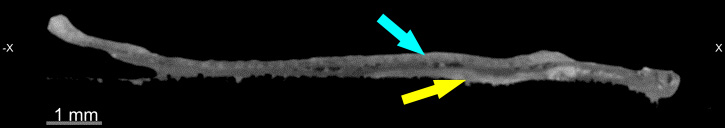

Supplement: Supplementary file 14 — Supplementary Figure S4c. [file 41598_2022_13907_MOESM14_ESM.jpg]

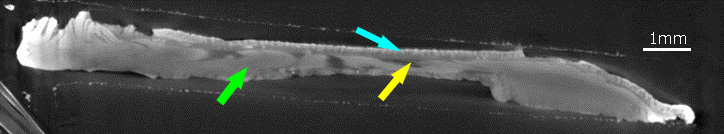

Supplement: Supplementary file 15 — Supplementary Figure S4d. [file 41598_2022_13907_MOESM15_ESM.jpg]

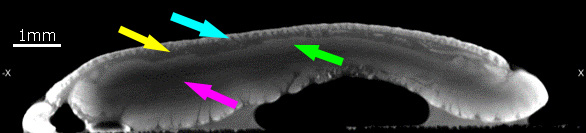

Supplement: Supplementary file 16 — Supplementary Figure S4e. [file 41598_2022_13907_MOESM16_ESM.jpg]

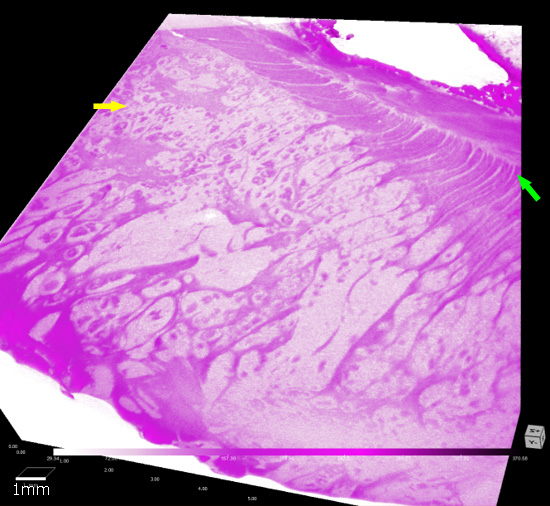

Supplement: Supplementary file 17 — Supplementary Figure S5a. [file 41598_2022_13907_MOESM17_ESM.jpg]

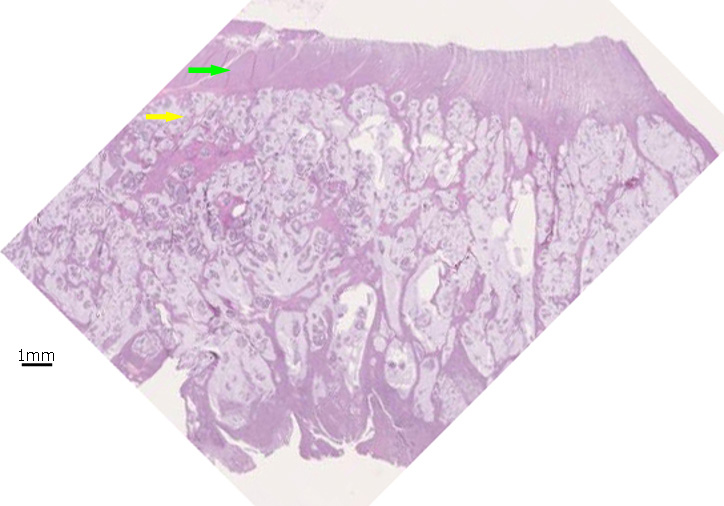

Supplement: Supplementary file 18 — Supplementary Figure S5b. [file 41598_2022_13907_MOESM18_ESM.jpg]
